# Sustainable Conversion of Pistachio Shells into Functional Biocarbons: Structural Evolution, Surface Properties, and Adsorptive Removal of Methyl Orange

**DOI:** 10.3390/ma19112231

**Published:** 2026-05-25

**Authors:** Barbara Charmas, Katarzyna Jedynak, Barbara Wawrzaszek, Lizaveta Tuflina

**Affiliations:** 1 Department of Chromatography, Faculty of Chemistry, Institute of Chemical Sciences, Maria Curie-Sklodowska University, Maria Curie-Sklodowska Sq. 3, 20-031 Lublin, Poland; barbara.wawrzaszek@mail.umcs.pl (B.W.); lizaveta.tuflina@gmail.com (L.T.); 2 Faculty of Exact and Natural Sciences, Institute of Chemistry, Jan Kochanowski University, Uniwersytecka Str. 7, 25-406 Kielce, Poland; katarzyna.jedynak@ujk.edu.pl

**Keywords:** pistachio-shell biocarbons, activated porous carbons, methyl orange adsorption, adsorption kinetics, waste biomass valorization

## Abstract

**Highlights:**

- Pistachio shells were converted into activated biocarbons with tunable porosity and surface chemistry;
- CO_2_ activation produced highly microporous carbons, while steam generated a more hierarchical pore network;
- Stronger activation rebuilt the carbon matrix, increased basicity, and reduced thermal stability;
- PM-8-H_2_O-2 showed the highest methyl orange uptake due to wider pores and higher external surface area;
- MO adsorption followed PSO kinetics and Freundlich behavior, with a spontaneous exothermic character.

**Abstract:**

This study aimed to produce biocarbons from pistachio shells and estimate the effect of physical activation with CO_2_ and overheated steam on their physicochemical, thermal, and adsorption properties in relation to methyl orange. Biocarbons were obtained by pyrolysis at 800 °C and subsequently activated under different conditions. From the results, the type of activating agent substantially determined the development of pore structure and surface chemistry. CO_2_ activation favored the formation of primarily microporous materials with a very large specific surface area, whereas steam activation led to a more open, hierarchical pore system with a greater pore volume and a larger contribution to external surface area. The most favorable textural properties were found for the samples PM-8-CO_2_-3 and PM-8-H_2_O-2. The FTIR, Raman, Boehm titration, CHN, SEM-EDS, and TG/DTG/DTA analyses confirmed that activation caused reconstruction of the carbon matrix, modification of the surface functional groups, and a decrease in thermal stability with increasing activation intensity. The adsorption studies proved that the sample PM-8-H_2_O-2 exhibited the largest efficiency in methyl orange removal. The adsorption kinetics were best described by the pseudo-second-order model, whereas the equilibrium data were best fitted by the Freundlich model. The adsorption process was spontaneous and exothermic.

## 1. Introduction

Escalating environmental problems include the contamination of water and soils by heavy metals and persistent organic compounds, the need to reduce waste generation and ensure sustainable resource management, and the need to develop materials derived from waste biomass [1,2,3,4,5,6,7,8,9]. In this context, biocarbons, defined as porous carbon materials obtained in the process of biomass pyrolysis under limited oxygen-access conditions, are of particular importance [10]. Due to their developed porous structure, the presence of surface functional groups, and the ability to modify textural and chemical properties, biocarbons are considered a promising material for environmental applications, including the adsorption of pollutants from aqueous solutions [11,12,13].

The properties of biocarbons depend primarily on the type of precursor, pyrolysis parameters, and activation method [14]. During the thermal treatment of biomass, depolymerization, fragmentation, and reorganization of the structure take place, leading to the formation of an aromatic carbon matrix and the development of porosity [15,16]. Properties such as the specific surface area, pore volume, and distribution, as well as the nature of the surface chemistry, are particularly important as they determine the ability of the material to adsorb impurities [17,18]. In order to improve these properties, activation processes can be used, among which physical activation, performed with the use of CO_2_ or steam, is particularly attractive from the perspective of green chemistry. Compared to chemical activation, it does not require the use of aggressive reactants or the generation of secondary waste that needs to be neutralized, and at the same time, it allows for effective development of the porous structure of the material [19,20].

Among lignocellulosic waste raw materials, pistachio shells are of significant interest, being a by-product of the agri-food industry, and may be efficiently processed into carbon materials [21,22]. Their chemical composition, as well as fibrous, rigid structure, make them promising precursors for the production of biocarbons with good adsorption properties [23,24]. The use of this type of waste is in line with the ideas of circular economy and sustainable development, as it allows for a simultaneous reduction in waste and for obtaining functional materials for environmental applications [25].

Despite the growing interest in biocarbons from waste biomass, there is still a need for research to explain how the type of activating agent affects the development of the porous structure, surface chemistry, thermal stability, and adsorption properties of materials simultaneously. This is particularly true for the adsorption of dyes, for which both the accessibility of pores and the nature of the adsorbent surface are important. In this aspect, the novelty of this study lies in its comprehensive and comparative evaluation of pistachio-shell-derived biocarbons, physically activated with two different activating agents—CO_2_ and superheated steam—under systematically varied activation conditions. Unlike studies focused mainly on the development of surface area, this work correlates the evolution of pore structure, surface functional groups, elemental composition, morphology, structural ordering, and thermal stability with the adsorption performance toward methyl orange. This approach makes it possible to distinguish the role of the highly microporous structures generated by CO_2_ activation from the more open, hierarchical pore network produced by steam activation. As a result, this study provides new insight into how the activation pathway controls the balance between textural development, surface chemistry, and dye adsorption efficiency in sustainable biocarbons obtained from pistachio shell waste.

This study aimed to obtain biocarbon from pistachio shells by pyrolysis, and then to modify it by physical activation using CO_2_ and water vapor. The obtained materials were subjected to comprehensive physicochemical, structural, and thermal characterization, and their adsorption properties in relation to methyl orange were estimated. The conducted research is of both academic importance, as it allows for a better understanding of the relationship between the method of activation and the properties of the material, and practical importance, indicating the possibility of designing effective and sustainable carbon adsorbents from lignocellulosic waste.

## 2. Materials and Methods

### 2.1. Biocarbon Preparation

**Scheme 1:**
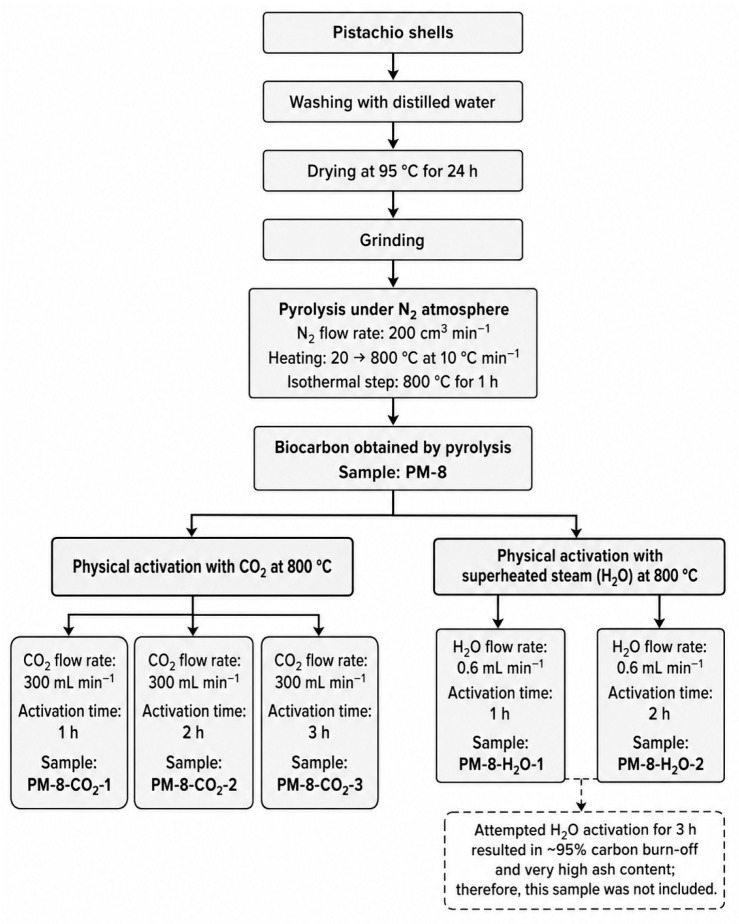
Flowchart of biocarbon preparation from pistachio shells.

Pistachio shells were washed with distilled water to remove impurities, after which the material was dried at 95 °C for 24 h and then ground. Pyrolysis was performed under a nitrogen atmosphere (flow rate: 200 cm^3^ min^−1^), whereas CO_2_ and H_2_O were used as physical activating agents. In the first stage, the raw precursor was heated from 20 to 800 °C at a rate of 10 °C min^−1^. After reaching the final temperature, the material was maintained at 800 °C in N_2_ for 1 h under isothermal conditions. The material obtained solely by pyrolysis was designated as PM-8. The PM-8 biocarbon was subsequently subjected to physical activation at 800 °C using CO_2_ and H_2_O. The CO_2_-activated samples (CO_2_ flow rate: 300 mL min^−1^) were denoted as PM-8-CO_2_-x, where x indicates the activation time of 1, 2, or 3 h. Similarly, the materials activated with superheated steam (H_2_O flow rate: 0.6 mL min^−1^) were designated as PM-8-H_2_O-x, where x denotes the activation time of 1 or 2 h. An attempt to extend the H_2_O activation time to 3 h resulted in a very large extent of carbon burn-off (~95%), and the resulting sample contained a very large amount of ash. The preparation procedure of pistachio-shell-derived biocarbons is summarized in Scheme 1.

### 2.2. Structural Characteristics

The porous structural parameters of the analyzed biocarbons were determined by low-temperature (−195.8 °C, degassing at 200 °C for 8 h) adsorption and desorption of nitrogen. The specific surface area of S_BET_ was calculated based on the Brunauer–Emmett–Teller equation (*S*_BET_) [26]. The total pore volume was determined at a relative pressure p/p_0_ close to 0.99. The proportion of micropores was estimated using the t-Plot method [27], while the pore radius was determined on the basis of the relationship *R*_av_ = 2*V*/*S*_BET_. The desorption data were used to calculate the pore-size distributions (PSDs, *f*_v_(*R*_p_) ~ d*V*_p_/d*R*_p_) based on the self-regularization procedure assuming the slit-shaped pore model [28,29].

### 2.3. SEM/EDS

Biocarbon morphology was determined using a scanning electron microscope (Quanta 3DFEGFEI, Field Electron and Ion Co., Hillsboro, OR, USA). The investigations were carried out without gold sputtering under low-vacuum conditions (a voltage of 5 kV). To perform qualitative and quantitative analyses, X-ray spectroscopy SEM/EDS (EDAX, Mahwah, NJ, USA) was used. The measurements were performed at an accelerating voltage of 20 kV.

### 2.4. CHN

The elemental analysis (CHN) was performed using a EuroEA Elemental Analyser (EuroVector, Milan, Italy). To ensure the accuracy of the results obtained, the samples were previously dried to a constant mass.

### 2.5. FTIR-ATR

FTIR-ATR measurements were made using the Perkin–Elmer Spectrum 400 FT-IR/FT-NIR spectrometer (Perkin–Elmer, Waltham, MA, USA) with a diamond chamber with total reflection (ATR). The samples were dried and powdered. All spectra were registered in the range of 4000–650 cm^−1^ with a resolution of 4 cm^−1^.

### 2.6. Raman

The Raman spectra were registered using a Raman Station 400 F (Perkin Elmer, Waltham, MA, USA) with a cooled CCD detector and a diode laser at a laser wavelength of 785 nm. Before the calculation of the D/G intensity ratio, the Raman spectra were subjected to baseline correction. The Raman signal in the 1000–1800 cm^−1^ region was then deconvoluted using the Lorentzian peak-fitting algorithm in Origin 8.6 software. The D band, located at approximately 1350 cm^−1^, and the G band, located at approximately 1580 cm^−1^, were fitted as individual components. The quality of fitting (*R*^2^) was verified by the agreement between the experimental spectrum and the cumulative fitted curve. The degree of carbon graphitization, *I*_D_/*I*_G_ ratio, was calculated from the intensities of the fitted D and G bands. In addition, the crystallinity of carbons was determined as *K* = 100·*I*_G_/(*I*_D_/*I*_G_).

### 2.7. Thermal Analysis

Thermal stability assessment of biocarbons was performed using a derivatograph (Derivatograph C, MOM, Budapest, Hungary). The samples (~10 mg) were placed in the corundum measuring crucibles and heated in an air atmosphere in the temperature range of 20–1000 °C (linear temperature increase: 10 °C min^−1^) using Al_2_O_3_ as a reference. The TG, DTG, and DTA curves were recorded. TG analysis was also performed in a nitrogen atmosphere (20–900 °C, 10 °C min^−1^) to approximate the volatile matter content (*VC*%), the mineral residue fraction (*A*%), and the bound carbon content (*FC*%).

### 2.8. Character of the Surface Using the Boehm Method

The content of surface groups of acid and base nature was determined by the Boehm method. Samples (~0.2 g) were flooded with 10 mL of hydrochloric acid solution (0.05 mol L^−1^) or 10 mL of NaOH (0.05 mol L^−1^). Suspensions were mixed in a shaker (Grant Instruments Ltd., Shepreth, UK, 25 °C, 140 rpm, 24 h) and then filtered using syringe filters (PTFE 30 mm, 0.45 µm, Chemland, Stargard, Poland). After dilution, filtrates were potentiometrically titrated (716 DMS Titrino, Metrohm, Herisau, Switzerland) with 0.05 mol L^−1^ HCl (to determine acidic groups) or 0.05 mol L^−1^ NaOH (to determine total basic groups). The tests were repeated 3 times.

### 2.9. Surface pH Measurement

The pH value of the biocarbons was investigated according to the procedure described in [30]. The dried biocarbons (~0.1 g) were mixed with 5 cm^3^ of redistilled water. Then, the samples were shaken (Grant Instruments Ltd., Shepreth, UK, 25 °C, 140 rpm, 24 h) and the pH of the solutions was measured. The tests were repeated 3 times.

### 2.10. Bulk Density Analysis

The bulk density of biocarbons was determined using the volume–mass method. About 3 cm^3^ of the sample was placed in a measuring cylinder, and then its mass was determined. The cylinder was repeatedly struck against a hard surface until the volume of the material stabilized, after which the volume of the sample was read. Each measurement was performed for five repetitions. The bulk density (*ρ*_b_) was calculated as the ratio of the mass of the sample (*m*_s_) to the volume occupied by the material (*V*_s_), according to the equation *ρ*_b_ = *m*_s_/*V*_s_, where *ρ*_b_ is the bulk density [g cm^−3^], *m*_s_ is the sample mass [g], and *V*_s_ is the sample volume [cm^3^].

### 2.11. Total Pore Volume

The total pore volume of the biocarbons, including micropores, mesopores, macropores, and intra-particle spaces, was determined by methanol titration. For each sample, five weights of approximately 0.05 g were prepared, and then methanol was dosed by droplets until a compact material structure was obtained. In order to facilitate the penetration of methanol into the porous system, measurements were made using ultrasound assistance. The total pore volume (*V*_total_) was calculated as the quotient of the volume of methanol (*V*_MeOH_) and the mass of the sample (*m*_s_), according to the equation *V*_total_ = *V*_MeOH_/*m*_s_, where *V*_total_ is the total pore volume [cm^3^ g^−1^], *V*_MeOH_ is the volume of methanol consumed during titration [cm^3^], and *m*_s_ is the mass of the sample [g].

### 2.12. Batch Adsorption Study

In the study, methyl orange (MO) was used as a model anionic dye. The adsorption process was carried out with the use of two selected adsorbents: PM-8-CO_2_-3 and PM-8-H_2_O-2. The selection of materials at this stage was based on the characteristics of their porous structure.

At the initial stage of the research, an analysis of the adsorption kinetics was performed. Measurements were made at a constant temperature of 303 K. For this purpose, methyl orange (MO) solutions were prepared with the initial concentrations of about 400 mg·L^−1^ for the PM-8-CO_2_-3 sample and about 800 mg·L^−1^ for PM-8-H_2_O-2. The phase contact time ranged from 30 to 4320 min.

Adsorption isotherms were determined under dynamic conditions at temperatures of 283 K, 293 K, and 303 K for MO solutions with concentrations of 100–1500 mg dm^−3^. Adsorption studies were carried out in 50 mL Erlenmeyer flasks in an incubator (Orbital Shaker—Incubator ES-20, Grant-Bio, Royston, UK) for a defined time. The studies were carried out using samples weighing 0.02 g, previously dried, which were placed in reaction flasks and then mixed with 10 cm^3^ of MO solution. The contact time of the solid phase with the solution was 720 min (PM-8-CO_2_-3) or 2880 min (PM-8-H_2_O-2). The equilibrium time was determined based on the preliminary studies of the adsorption process kinetics. After the process, the samples were filtered with syringe filters (PTFE 30 mm, 0.45 μm), and then the equilibrium concentration of the dye in the solution was determined by UV–Vis spectrophotometry (UV–Vis spectrophotometer Helios Gamma, Spectro-Lab, Łomianki, Poland) at a wavelength of λ—464 nm. It was found that the syringe filters do not adsorb MO.

## 3. Results and Discussion

**Figure 1:**
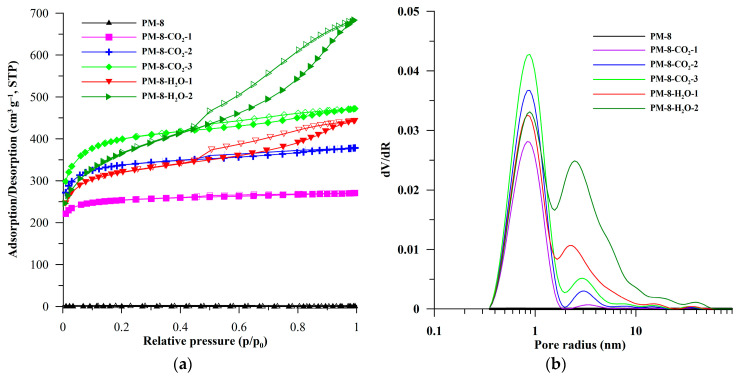
(**a**) Adsorption/desorption isotherms of N_2_ at 77 K (full symbols—adsorption, empty symbols—desorption) and (**b**) pore-size distributions for the tested biocarbons.

Figure 1 presents the N_2_ adsorption/desorption isotherms (Figure 1a) and the pore-size distributions (PSDs, Figure 1b) for the initial unactivated biocarbon and the materials physically activated with CO_2_ and H_2_O, while Table 1 summarizes their structural parameters. The application of physical activation leads to a pronounced reconstruction of the porous structure, and the nature of these changes depends significantly on both the type of activating agent and the process duration. The initial PM-8 material exhibits virtually no developed texture, as evidenced by its very low specific surface area (*S*_BET_ = 0.5 m^2^ g^−1^; Table 1). This indicates that the substantial development of specific surface area and porosity occurs only during the activation stage. The analysis of the curves shown in Figure 1, supported by the data compiled in Table 1, demonstrates that the samples activated with CO_2_ and H_2_O differ not only in the degree of surface development but, above all, in the contribution of micropores and wider pores to the overall porous structure.

For the CO_2_-activated samples, the shape of the isotherms indicates a dominant contribution of micropores, as evidenced by the sharp increase in adsorption at low relative pressures (Figure 1a). In particular, for the sample PM-8-CO_2_-1, the rapid attainment of a near-plateau state suggests the predominance of narrow micropores and a limited contribution of larger transport pores. With increasing activation time, a systematic increase in the total adsorption is observed, which is directly reflected in the increase in *S*_BET_ from 974 to 1478 m^2^ g^−1^ and in *V*_p_ from 0.419 to 0.730 cm^3^ g^−1^ (Table 1). At the same time, the contribution of micropore surface area decreases from 86 to 71%, whereas the external surface area (*S*_ext_) increases from 131 to 422 m^2^ g^−1^. These changes are accompanied by an increase in the meso- and macropore volumes from 0.084 to 0.298 cm^3^ g^−1^, as well as by a slight increase in the average pore radius from 0.86 to 0.99 nm. These results indicate that CO_2_ activation proceeds in a relatively mild and selective manner, leading primarily to the development of the existing microporosity and gradual broadening of the pores, without abrupt reconstruction of the entire material structure. This interpretation is supported by the pore-size distributions, which for this biocarbon series exhibit predominance of narrow pores and only a gradual broadening of distribution toward larger radii with increasing activation time (Figure 1b).

A distinctly different pattern of structural changes is observed for the biocarbons activated with superheated steam. Even the sample PM-8-H_2_O-1 is characterized by a large specific surface area (1177 m^2^ g^−1^), as well as by a greater contribution of external surface area (323 m^2^ g^−1^) and a smaller share of micropore surface area than in the corresponding samples from the CO_2_-activated series (Table 1). This suggests that H_2_O activation promotes not only the formation of micropores from the outset, but also a more intensive development of the structure and opening of wider pores. This effect is particularly evident for the sample PM-8-H_2_O-2, for which, despite the maintenance of a large specific surface area (1317 m^2^ g^−1^), a fundamental change in the character of porosity is observed. The contribution of micropore surface area decreases to 39%, whereas *S*_ext_ increases to 804 m^2^ g^−1^. At the same time, the total pore volume reaches the highest value among all analyzed samples (1.057 cm^3^ g^−1^), and the meso- and macropore volume is as high as 0.845 cm^3^ g^−1^ (Table 1). The accompanying increase in the average pore radius to 1.60 nm confirms intensive broadening of the porous structure. The shape of the isotherms for this series—including higher adsorption values over the entire p/p_0_ range, the absence of a distinct plateau, and a clearly developed hysteresis loop—indicates a significant contribution of mesopores and a more complex pore-system geometry (Figure 1a). This is also consistent with the character of the pore-size distributions, which suggest a broader and more heterogeneous pore system than that observed for the CO_2_-activated materials (Figure 1b).

Comparison of the two series clearly demonstrates that CO_2_ and H_2_O resulted in the formation of different surface structures. CO_2_ activation promotes the formation of materials with a very large specific surface area and dominant microporosity, whereas steam activation causes a more profound reconstruction of the carbon matrix, resulting in materials with a larger pore volume, a greater contribution of external surface area, and more developed meso- and macroporosity. This difference is clearly visible when comparing the samples PM-8-CO_2_-3 and PM-8-H_2_O-2. Despite their similar *S*_BET_ values, the former retains a distinctly microporous character, whereas the latter represents a more open and hierarchical structure. This indicates that the specific surface area alone is not sufficient for complete characterization of the material, and that the pore-size distribution, as well as the relationship between the micropore surface area and the external surface area, are also of key importance.

Additional evidence of the progressive development of porosity is provided by a decrease in bulk density with increasing extent of activation. In the CO_2_-activated series, the ρ_b_ value decreases from 0.5219 to 0.3898 g cm^−3^, whereas for the sample PM-8-H_2_O-2, it reaches 0.2569 g cm^−3^ (Table 1). This trend reflects an increasing fraction of void spaces within the material and a greater loosening of the structure, which is particularly pronounced in the case of steam activation.

From the standpoint of adsorption applications, the obtained results indicate that the textural parameters of the investigated biocarbons can promote different transport mechanisms and pore-space accessibility; however, a full assessment of their sorption capacities requires consideration not only of surface development and pore-size distribution, but also of surface chemistry and the nature of adsorbate–adsorbent interactions.

**Figure 2:**
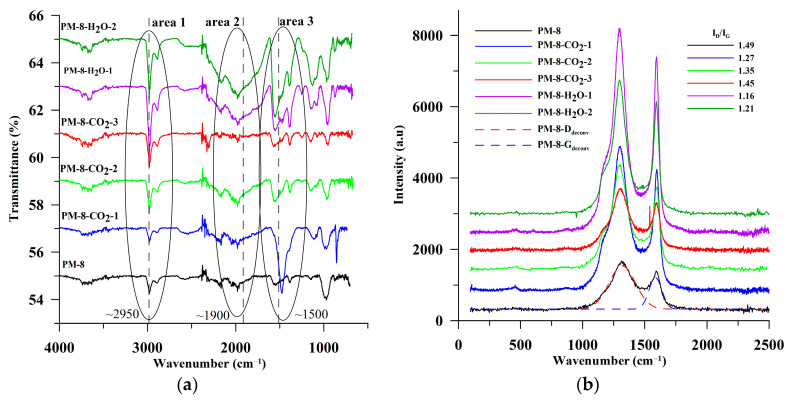
(**a**) FTIR and (**b**) Raman spectra of the tested biocarbons.

The content of surface functional groups was evaluated, among other methods, on the basis of FTIR spectral analysis (Figure 2a), which demonstrated that the materials retained a predominantly aromatic structure, characteristic of biocarbons and other carbonaceous materials. The spectra also confirm that both the type of activating agent and the treatment duration influenced the nature of the surface oxygen-containing groups. The broad, weakly defined band in the 3600–3200 cm^−1^ region can be assigned to the stretching vibrations of –OH groups originating from the phenolic hydroxyl groups, carboxylic groups, and surface-bound water. In turn, the bands in the 2950–2850 cm^−1^ region (Figure 2a, area 1) correspond to the stretching vibrations of aliphatic C–H bonds, indicating the presence of residual aliphatic or oxygen-containing aliphatic moieties in the structure of the investigated biocarbons. In the 1750–1500 cm^−1^ range, overlapping signals associated with C=O carbonyl vibrations and C=C skeletal vibrations of aromatic systems are observed (Figure 2a, area 2). The absence of a very intense, sharp band around 1700–1725 cm^−1^ suggests that free carboxylic groups do not dominate the material surface; instead, more conjugated oxygen-containing functionalities, such as ketones, quinones, or lactones associated with the aromatic carbon matrix, can be present. Such an interpretation is typical of materials with a substantial degree of carbonization, in which the contribution of condensed aromatic structures increases, whereas more labile oxygen-containing groups are partially eliminated.

The most pronounced differences among the investigated materials are observed in the 1300–1000 cm^−1^ region (Figure 2a, area 3), which is assigned to C–O vibrations in phenolic, ether, ester, and lactone groups. These bands are somewhat more distinct for the steam-activated samples, particularly after longer treatment times, indicating that H_2_O activation induces a stronger chemical reconstruction of the surface layer. In combination with the Boehm titration results (Table 2), such surface restructuring can more likely be attributed to an increase in the contribution of phenolic, ether, lactone, or other weakly acidic functionalities, accompanied by a reduced contribution of carboxylic groups. In the case of the CO_2_-activated samples, the signals in this region are weaker, and for the longest activation time, the spectrum becomes more flattened, which can be interpreted as a result of further aromatization and condensation of the carbon structure, together with the partial elimination of less stable oxygen-containing moieties. This trend is consistent with the literature data [31], according to which increasing the thermal treatment intensity promotes a reduction in the contribution of O–H, C=O, and C–O groups, while increasing the degree of ordering of the carbon matrix. The signals below 900 cm^−1^, in turn, can be attributed to out-of-plane C–H vibrations in aromatic rings, which further confirms the presence of condensed aromatic systems in the investigated materials [32].

The change in solution pH after contact with the investigated biocarbons indicates the presence of surface functional groups as well as mineral constituents. The high pH of the solution (approximately 10; Table 2) reflects the distinctly basic character of the adsorbent surface. This effect can be associated with both the predominance of basic surface groups and the contribution of mineral components, such as metal oxides and hydroxides, remaining after the thermal treatment of biomass. The gradual increase in pH with increasing activation time can indicate an increasing contribution of basic components in the analyzed materials. Although a high pH does not exclude the presence of acidic groups, it suggests that their contribution is relatively small. The data presented in Table 2 show that biocarbon activation leads to an evident increase in the number of basic groups with increasing activation time compared with the initial biocarbon, with superheated steam activation being more effective in this respect. The basic surface character can be of significant importance for adsorption processes as it promotes interactions with acidic or positively charged species, while simultaneously, it can confine the sorption of anions because of unfavorable electrostatic interactions.

The CHN elemental analysis demonstrated that the elemental composition of the investigated biocarbons changed markedly depending on the activation method (Table 3). The initial PM-8 sample was characterized by the largest mean carbon content (95.98%) and the mean nitrogen content (0.80%), while exhibiting a very small mean hydrogen content (0.10%). Activation with CO_2_ for 3 h reduced the mean carbon content to 94.44% and the mean nitrogen content to 0.43%, accompanied by only a slight increase in the mean hydrogen content to 0.13%. Even more pronounced changes were observed for the sample activated with steam for 2 h, for which the mean carbon content decreased to 91.63% and the mean nitrogen content to 0.11%, whereas the mean hydrogen content increased to 0.41%. These results confirm that activation, particularly with steam, promotes a more profound reconstruction of the material structure, associated with the partial removal of carbonaceous matter and a change in the chemical character of the surface layer.

The increase in hydrogen content observed during activation, accompanied by a simultaneous decrease in nitrogen content, can be explained by gasification reactions and reconstruction of biocarbon surface chemistry. Under the activation conditions, particularly in the presence of steam, the carbon matrix undergoes preferential removal in the form of CO and CO_2_, which reduces the percentage contribution of carbon and can result in a relative increase in hydrogen content. At the same time, oxygen–hydrogen-containing functionalities can be formed or exposed on the surface; however, the FTIR data and the Boehm titration results indicate that these are not strongly acidic carboxylic groups, but rather chemically more diverse oxygen-containing species. The pronounced decrease in nitrogen content can, in turn, be associated with the thermal degradation and transformation of nitrogen-containing functionalities into volatile products, mainly NH_3_, HCN, and N_2_. The accessibility of hydrogen radicals derived from H_2_O can further facilitate nitrogen transformations leading to NH_3_ release; therefore, steam activation results in a greater depletion of nitrogen in the material than CO_2_ activation (Table 3). Thus, the obtained results indicate that steam activation induces more profound reconstruction of both the carbon framework and the surface chemistry of the investigated materials.

The results of SEM-EDS (Table 4) confirm that carbon remains a dominant element in all tested samples, but its share decreases with increasing activation intensity, particularly in the case of vapor-activated materials. The C content decreases from 92.96% for the PM-8 sample to 85.84% for PM-8-H_2_O-2, accompanied by an increase in the oxygen content from 4.45 to 7.63%, both expressed by weight. This trend indicates that more intensive activation leads to partial oxidation of the surface and an increase in the proportion of aerobic surface structures. In the CO_2_-activated series, these changes are moderate for the PM-8-CO_2_-1 and PM-8-CO_2_-2 samples, while they are more pronounced for PM-8-CO_2_-3.

Activation also affects the relative contribution of mineral constituents. As the process progresses, the sum of elements present in trace amounts (excluding C and O) at contents both above 0.1% (Table 4, a *) and below 0.1% (Table 4, b *) increases, reaching the highest values for the sample PM-8-H_2_O-2, which indicates a greater contribution of inorganic phases in the material. A particularly evident trend is the increase in the Ca content in the more strongly activated samples, whereas the contents of Na and Cl decrease gradually. These results indicate that activation, especially with steam, leads not only to changes within the carbon matrix but also to relative enrichment of the surface in the mineral components, which can significantly affect the surface and adsorption properties of the investigated biocarbons.

Structural ordering of the biocarbons was estimated on the basis of Raman spectral analysis (Figure 2b). For all investigated biocarbons, the spectra exhibited two characteristic bands: the D band, located near 1350 cm^−1^, and the G band, appearing at approximately 1580 cm^−1^ (Table 5). After the baseline correction, the broad Raman signal in the 1000–1800 cm^−1^ region was deconvoluted using the Lorentzian peak-fitting algorithm (Figure 2b, dashed curves). This algorithm was selected because of a better fit (*R*^2^). The D and G bands were fitted as individual components. The quality of fitting was verified by the agreement between the experimental spectrum and the cumulative fitted curve (Table 5, *R*^2^). The *I*_D_/*I*_G_ ratio was calculated from the intensities of the fitted D and G bands. The G band is associated with vibrations of carbon atoms in the ordered aromatic structures with sp^2^ hybridization, whereas the D band reflects the presence of structural defects, disorder, and edges of small aromatic domains. The presence of both bands indicates that the investigated materials possess a partially ordered structure while at the same time containing numerous disturbances typical of biocarbons and activated carbonaceous materials [12].

As follows from the comparison of the spectra (Figure 2b), activation affects the structure of the carbon matrix; however, these changes do not represent a complete reconstruction toward well-crystallized graphitic carbon. The *I*_D_/*I*_G_ ratio values fall within the relatively narrow range of 1.21–1.49, indicating that all biocarbons are characterized by numerous structural defects and retain a turbostratic character. At the same time, the improved separation of the D and G bands after activation suggests partial ordering of local aromatic domains. Thus, the activation process does not eliminate the structural defects of biocarbons, but rather leads to structural rearrangement: on the one hand, less ordered carbonaceous fragments are removed, while on the other hand, more condensed aromatic systems are exposed or formed.

Against this background, the CO_2_- and H_2_O-activated samples exhibit somewhat different patterns of change. For the CO_2_-activated series, the I_D_/I_G_ values are slightly higher, which can indicate the persistence of a greater contribution of structural defects or smaller aromatic domains. In comparison, the steam-activated samples display slightly smaller values of this parameter and more distinct separation of the bands, suggesting better ordering of the local carbon structure. The values of the *K* coefficient (Table 5), calculated as the relative share of the G-band in the total intensity of the D and G bands, are in the range of 40.8–46.2%, which indicates a moderate and similar degree of ordering of the carbon structure of the tested biocarbons. The presence of intense D and G bands confirms that the analyzed materials have a turbostratic structure typical of biocarbons, consisting of small aromatic domains sp^2^, numerous structural defects, and less ordered areas.

When considered together with the FTIR results, the Raman analysis confirms that activation simultaneously induces changes in both the carbon framework and the surface chemistry, while also indicating partial ordering of local aromatic domains accompanied by the retention of a large extent of defectiveness. This is consistent with the FTIR findings, which show that this process is accompanied by transformations of oxygen-containing functionalities. Overall, it can be concluded that activation does not lead to simple “graphitization” of the material, but rather to a complex structural reconstruction involving both the reorganization of aromatic domains and changes in surface functional groups.

**Figure 3:**
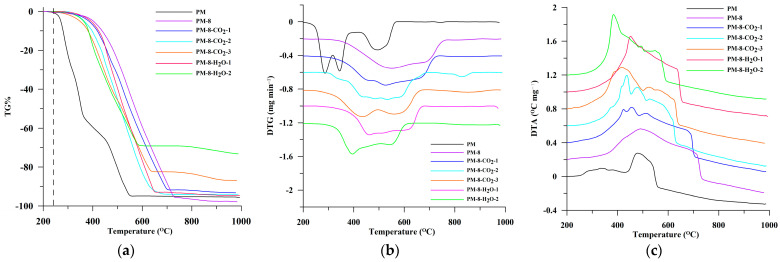
Course of the (**a**) TG%, (**b**) DTG, and (**c**) DTA curves for the obtained biocarbons.

The observed changes in the local ordering of aromatic domains affect the thermal stability of the materials after activation; as porosity develops simultaneously, the contribution of edge defects and the mineral fraction increases, and the matrix becomes more reactive. Figure 3 presents the TG% curves (Figure 3a), DTG curves (Figure 3b), and DTA curves (Figure 3c) determined for the investigated biocarbons.

The TG/DTG/DTA profiles (Figure 3) are consistent with the proximate analysis results (Table 6), and confirm that the thermal stability of the biocarbons decreases with increasing activation intensity. PM-8 exhibits the decomposition profile most strongly shifted towards higher temperatures and the lowest rate of mass loss, confirming its largest thermal resistance. In the CO_2_- and H_2_O-activated samples, the main decomposition stage becomes more pronounced and extends over a broader temperature range, while the minima in the DTG curves become deeper, indicating increased reactivity and greater structural heterogeneity of the activated biocarbons. The strongest effect is observed for PM-8-H_2_O-2, which is associated with profound reconstruction of the carbon framework and relative enrichment of the material in the mineral fraction. These results show that the development of the porous structure as a result of activation occurs at the expense of the degree of condensation of the carbon matrix, leading to a reduction in its thermal resistance. This is consistent with the conclusions drawn from the Raman analysis, which indicate only a partial development of locally ordered aromatic domains rather than a pronounced increase in structural ordering and, thus, in the thermal stability of the materials.

The results of the thermal analysis (Figure 3) and proximate analysis (Table 6) further indicate that the method and intensity of activation affect both the composition and the thermal stability of the investigated biocarbons significantly. The non-activated PM-8 sample was characterized by the largest fixed-carbon content (%*FC* = 89.82%) and the smallest volatile matter content (%*VC* = 5.75%), indicating a great extent of carbonization and the largest thermal stability. Activation resulted in an increase in the proportion of volatile matter and ash, accompanied by a decrease in the fixed-carbon fraction. This effect became more pronounced with increasing process intensity. In the CO_2_-activated series, a gradual increase in %*VC* and a decrease in %*FC* were observed, indicating progressive reconstruction and partial gasification of the carbon matrix. Even more pronounced changes were found for the steam-activated samples, especially PM-8-H_2_O-2, which exhibited the largest volatile matter content (%*VC* = 20.54%), the smallest fixed-carbon content (%*FC* = 52.8%), and the largest ash content (%*A* = 26.66%), confirming the highest degree of structural transformation. The significantly higher ash content in the H_2_O-activated samples, despite the use of the same precursor, is due to the more intense nature of steam activation compared to CO_2_ activation. The presented results clearly indicate that water vapor causes stronger gasification and thermal degradation of the carbon matrix, which leads to a significant loss of organic/carbon fraction and relative enrichment of the mineral residue. This is confirmed by the results of TG and proximate analyses, as well as by the results of CHN and SEM-EDS, indicating a decrease in the carbon content and a relative increase in the proportion of minerals after intensive steam activation.

**Figure 4:**
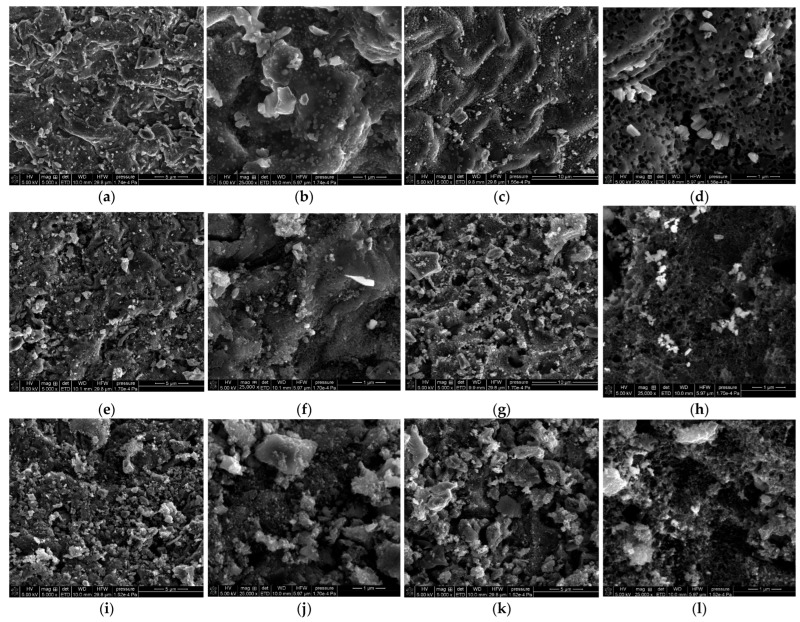
SEM images of (**a**,**b**) PM-8, (**c**,**d**) PM-8-CO_2_-1, (**e**,**f**) PM-8-CO_2_-2, (**g**,**h**) PM-8-CO_2_-3, (**i**,**j**) PM-8-H_2_O-1, and (**k**,**l**) PM-8-H_2_O-2 samples (magnifications: ×5000 (**a**,**c**,**e**,**g**,**i**,**k**) and ×25,000 (**b**,**d**,**f**,**h**,**j**,**l**)).

The SEM images (Figure 4) revealed a pronounced effect of physical activation on the morphology of the investigated biocarbons. The initial PM-8 sample (Figure 4a,b) is characterized by a relatively compact and poorly developed surface, with a limited number of open voids and a relatively continuous carbon matrix. The visible surface irregularities are local in nature, whereas the overall morphology indicates a small extent of porous texture development. With activation, a gradual reconstruction of the structure is observed, involving increased roughness, development of irregular depressions, and pore opening.

In the CO_2_-activated series, the morphological changes become more pronounced with increasing process time. For the sample PM-8-CO_2_-1 (Figure 4c,d), the surface is already markedly more corrugated and perforated than that of the initial material, indicating an initial intensive stage of carbon matrix etching. In the sample PM-8-CO_2_-2 (Figure 4e,f), this effect becomes more pronounced, and the surface becomes rougher and more heterogeneous, with a greater number of voids and depressions. The most advanced transformations in this series were observed in PM-8-CO_2_-3 (Figure 4g,h), which exhibits a largely developed, irregular morphology with numerous material losses and a clearly degraded carbon framework. This indicates that prolonging the CO_2_ activation time results in the successive development of porosity and simultaneously progressive erosion of the carbon walls. The steam-activated samples exhibit even more pronounced morphological changes. For PM-8-H_2_O-1 (Figure 4i,j), the surface is more heterogeneous, fragmented, and granular than that observed for the corresponding CO_2_-activated samples. In turn, PM-8-H_2_O-2 (Figure 4k,l) is characterized by the most extensively transformed structure among all the investigated materials, displaying a distinctly sponge-like, largely perforated, and degraded morphology. The presence of numerous voids, irregular cavities, and a fine-grained texture indicates intensive gasification of the carbon matrix and advanced opening of the structure.

The obtained results confirm that both CO_2_ and H_2_O develop the surface morphology of biocarbon effectively; however, steam acts considerably more aggressively, leading to stronger fragmentation and greater degradation of the carbon framework. Overall, activation induced transition from a compact structure to one that became increasingly rough, porous, and heterogeneous, which is characteristic of the progressive removal of the more reactive fragments of the carbon phase and the generation of a new, more developed surface.

### 3.1. Adsorption Studies

**Figure 5:**
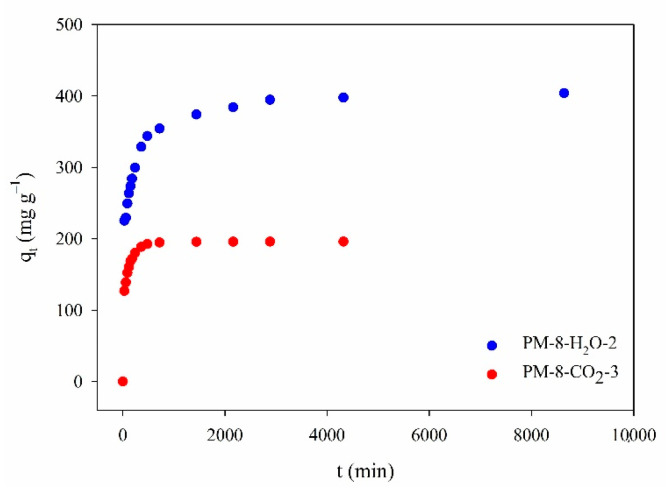
Experimental kinetic data of MO on the studied biocarbons of the initial concentration of 400 mg L^−1^ (PM-8-CO_2_-3) and of 800 mg L^−1^ (PM-8-H_2_O-2).

The main objective of the preliminary adsorption studies was to estimate the effect of different types of physical activation of biocarbons (CO_2_ and H_2_O) on their capacity in the case of methyl orange adsorption. This method is widely used in the effective treatment of air and water. Methyl orange (MO) is an azo anionic dye. It belongs to one of the most important classes of synthetic organic dyes because of its vivid color, durability, chemical stability, and ease of application [33]. MO is used in paper, textile, printing, pharmaceutical, food, biomedical, chemical, and technological industries. Figure 5 presents the experimental kinetic data for the selected materials. The adsorption equilibrium state was reached after 720 min and 2880 min for PM-8-CO_2_-3 and PM-8-H_2_O-2, respectively. A longer time was required to reach equilibrium for PM-8-H_2_O-2 (48 h) compared to PM-8-CO_2_-3 (12 h), which can be attributed to its more developed porous structure and larger pore volume, extending the diffusion path of the dye molecules.

The different initial MO concentrations used in kinetic studies for the PM-8-CO_2_-3 (400 mg L^−1^) and PM-8-H_2_O-2 (800 mg L^−1^) samples were due to their markedly different adsorption capacities, as determined by initial adsorption tests. The PM-8-H_2_O-2 sample, due to its more open, hierarchical porous structure, larger total pore volume, and greater proportion of outer surface, showed a much greater ability to remove methyl orange. Preliminary experiments conducted on this material at a lower concentration of dye (400 mg L^−1^) showed nearly complete decolorization of the solution; therefore, in further studies for this sample, an MO solution with a higher initial concentration was used. This allowed for more reliable kinetic data and for a better assessment of the actual adsorption capacity of the material. For PM-8-CO_2_-3, a lower initial concentration was sufficient because the sample showed a lower adsorption capacity and a more microporous character. Therefore, the results presented in Figure 5 should be considered as an assessment of the adsorption kinetics under conditions selected for the properties of individual adsorbents, and not as a direct comparison carried out at an identical initial concentration of the dye.

**Figure 6:**
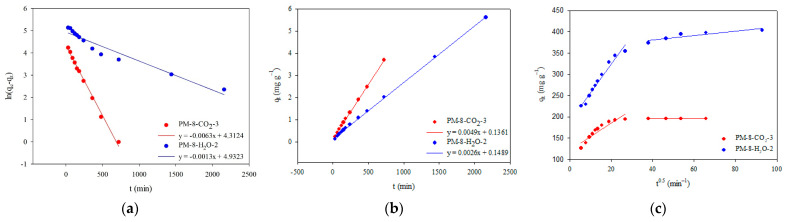
Linear fits to the (**a**) pseudo-first-order PFO, (**b**) pseudo-second-order PSO, as well as (**c**) intra-particle diffusion IPD models.

To describe the kinetics of MO adsorption on the studied biocarbons, the linear forms of the pseudo-first-order model (PFO, called the Lagergren equation [34]) and of the pseudo-second-order model (PSO, Ho equation [35]) as well as the Weber–Morris intra-particle diffusion (IPD) model [36] were applied (Table 6). Figure 6a–c present the adjustment of the experimental data of MO adsorption on the tested biocarbons to the applied kinetic models. The kinetic parameters calculated based on the relevant equations are collected in Table 7.

The kinetics of MO adsorption (Figure 6b) are best described by the pseudo-second-order (PSO) model, as evidenced by the high coefficients of determination (*R*^2^ ≈ 0.999, Table 7) obtained for both adsorbents. However, the best model fit does not determine the adsorption mechanism unambiguously. The adsorption capacities calculated from the PSO model (Table 7) are consistent with the experimental values (*q*_e,exp_), which confirms the adequacy of the model fit. Application of the intra-particle diffusion (IPD) model showed that the adsorption process proceeds in two stages. The first stage is associated with intra-particle diffusion, whereas the second corresponds to the attainment of equilibrium. The nonzero and positive values of the intercept (*C*) suggest that pore diffusion is not the only rate-limiting step, and that boundary layer diffusion also plays a significant role.

### 3.2. Adsorption Isotherms

The Langmuir [37], Freundlich [38], Langmuir–Freundlich [39], and Temkin [40] models were applied to interpret the experimental adsorption isotherms. The nonlinear regression model using Origin Microcal 10 (together with the Levenberg–Marquardt algorithm) was applied to assign an appropriate adsorption model and to calculate sorption parameters based on each of the models.

Langmuir isotherm parameters *q*_m_ and *K*_L_ were calculated based on Equation (1) [37]:

(1) $$q_{e}=\frac{q_{m}K_{L}C_{e}}{1+K_{L}C_{e}}\;$$

where *q*_m_ is the maximum adsorption capacity corresponding to the total monolayer coverage on the adsorbent surface (mg g^−1^); *K*_L_ is the Langmuir constant (L g^−1^).

The relationship of the experimental data *q*_e_ vs. *C*_e_ was also analyzed based on the Freundlich model (Equation (2), [38]):

(2) $$q_{e}=K_{F}C_{e}^{1/n}$$

where *K*_F_ is the Freundlich isotherm constant (mg^(1 − 1/n)^ (L)^1/n^ g^−1^); *n* is the empirical constant describing the heterogeneity of the adsorbent surface.

The Langmuir–Freundlich (Sips) isotherm is presented in the form of Equation (3) [39]:

(3) $$q_{m}=\frac{q_{m}{{(K}_{LF}C_{e})}^{n}}{1+{{(K}_{LF}C_{e})}^{n}}$$

where *K*_LF_ is the Langmuir–Freundlich constant (L mg^−1^); *n* is the constant.

The Temkin isotherm is presented in the form of Equation (4) [40]:

(4) $$q_{e}=\frac{RT}{b_{T}}ln(K_{T}C_{e})$$

where *R* is the gas constant (8.314 J mol^−1^ K^−1^); *T* is the absolute temperature (K); *b*_T_ (J mol^–1^ g mg^−1^) and *K*_T_ (L mg^−1^) are the Temkin constants.

**Figure 7:**
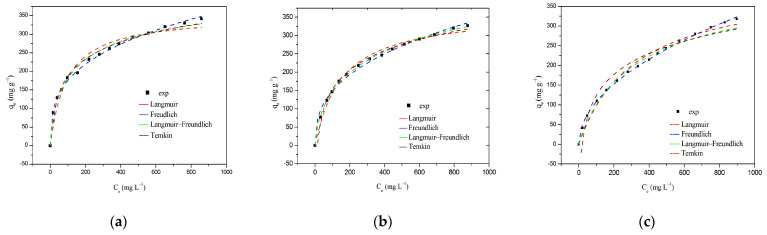
Fitting the isotherms models to the experimental equilibrium data for PM-8-CO_2_-3 at (**a**) 10 °C, (**b**) 20 °C, and (**c**) 30 °C.

**Figure 8:**
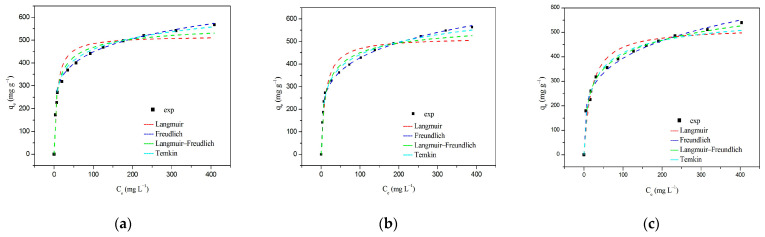
Fitting the isotherms models to the experimental equilibrium data for PM-8-H_2_O-2 at (**a**) 10 °C, (**b**) 20 °C, and (**c**) 30 °C.

The equilibrium experimental data determined at 10, 20, and 30 °C (black squares), together with the nonlinear fits to the applied isotherm models, are presented in Figure 7a–c for PM-8-CO_2_-3 and Figure 8a–c for PM-8-H_2_O-2, while the derived parameters are summarized in Table 7. The best fit was obtained for the Freundlich model (*R*^2^ > 0.99), indicating the heterogeneous surface character and the possibility of multilayer adsorption. The heterogeneity parameter values (*n* < 1) indicate strong interactions between the adsorbent and the adsorbate. Moreover, the n parameter can also indicate a substantial contribution of chemisorption to the process under study. The systematic increase in n values, from 0.317 to 0.497 for PM-8-CO_2_-3 and from 0.209 to 0.239 for PM-8-H_2_O-2 (Table 8), is associated with intensified interactions between the MO molecules and the biocarbon surface [41]. However, the superior fit of the Freundlich model suggests that the adsorption process is not limited to monolayer formation, but rather occurs on a surface with a non-uniform energy distribution.

The Langmuir isotherm model data (*q*_m_, Table 8) confirm large maximum adsorption capacity, ranging from 353.10 to 413.92 mg g^−1^ for PM-8-CO_2_-3 and from 519.40 to 521.18 mg g^−1^ for PM-8-H_2_O-2. The experimentally obtained values are slightly higher, indicating a further increase in the adsorption capability of these materials, and amount to 318.11–342.02 mg g^−1^ and 540.02–567.33 mg g^−1^, respectively.

### 3.3. Adsorption Thermodynamics

To fully characterize the nature of the adsorption process, thermodynamic parameters describing its course were determined, including the Gibbs free-energy change (Δ*G*, Equation (5)), enthalpy change (Δ*H*), and entropy change (Δ*S*, Equation (6)) [42].

(5) $$\Delta G{}^{\circ}=-RT\ln K_{L}$$

(6) $$\ln K_{L}=-\frac{∆H{}^{\circ}}{R}\times \frac{1}{T}+\frac{∆S{}^{\circ}}{R}$$

**Figure 9:**
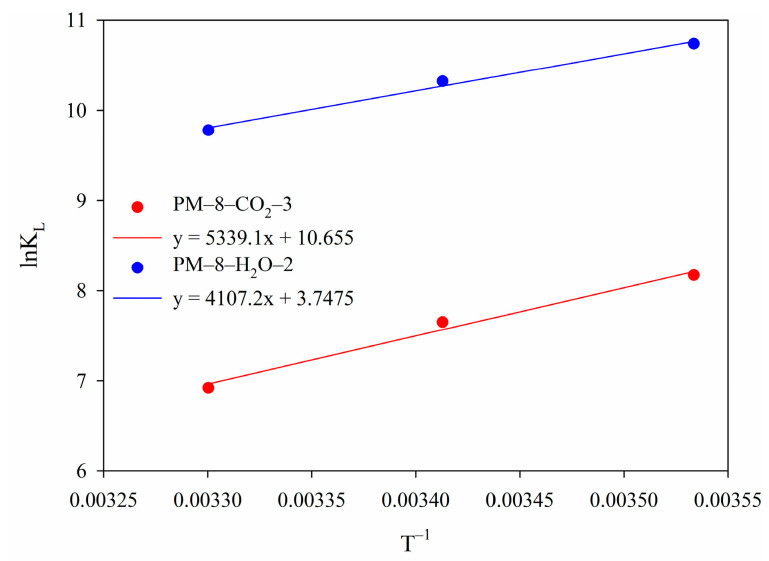
Plot ln*K*_L_ = f(*T*^−1^) for the tested biocarbons.

The dependence ln*K*_L_ = f(1/*T*) for all investigated adsorbents is given in Figure 9. The calculated values of Δ*G*°, Δ*H*°, and Δ*S*° are listed in Table 9.

The determined thermodynamic parameters of adsorption on the investigated biocarbons indicate that the process is exothermic, as confirmed by the negative enthalpy change values (Δ*H*, Table 9). For the CO_2_-activated material, Δ*H* was −44.4 kJ·mol**^−^**^1^, whereas for the steam-activated biocarbon (H_2_O), Δ*H* was −34.1 kJ·mol^−1^, suggesting stronger adsorbate–adsorbent interactions in the former material. The higher absolute value of Δ*H* for the CO_2_-activated sample can be attributed to its more developed microporous structure and greater surface heterogeneity, which promotes the presence of energetically favorable adsorption sites.

In both cases, negative entropy change values (Δ*S*, Table 9) were observed, amounting to −88.6 J·mol^−1^·K^−1^ for CO_2_ activation and −31.2 J·mol^−1^·K^−1^ for H_2_O activation, indicating a decrease in the extent of disorder at the solid–solution interface during adsorption. This phenomenon can be associated with the restricted mobility of adsorbate molecules, resulting from their confinement within the porous structure of the adsorbent. The markedly greater decrease in entropy for the CO_2_-activated biocarbon suggests a more restrictive adsorption environment and can be attributed to the presence of narrower micropores.

As follows from the thermodynamic analysis, the adsorption process is predominantly physisorptive in nature, with a possible contribution of weak specific interactions. Moreover, the negative Δ*S* values suggest that increasing temperature can affect adsorption efficiency adversely, indicating that the process is thermodynamically more favorable at lower temperatures. The negative Gibbs free-energy change values (Δ*G*) confirm the spontaneous nature of the adsorption process. The more negative values observed for PM-8-H_2_O-2 indicate its greater affinity for methyl orange.

### 3.4. Mechanism of MO Adsorption and Changes in Surface Chemistry

Based on the analysis of the porous structure (Table 1) and surface chemistry (Table 2), it can be concluded that methyl orange adsorption is a complex process resulting from the synergistic action of several factors: textural properties, in which micropores provide adsorption sites while mesopores facilitate mass transport and improve the accessibility of these sites; surface chemistry, where the presence of basic functional groups promotes electrostatic interactions with the anionic dye; and adsorbate properties, since the molecular size of methyl orange promotes adsorption on the materials with well-developed mesoporosity. As a result, the PM-8-H_2_O-2 sample exhibits the largest adsorption efficiency, owing to the optimal combination of large pore volume, well-developed mesoporosity, and strongly basic surface character.

**Figure 10:**
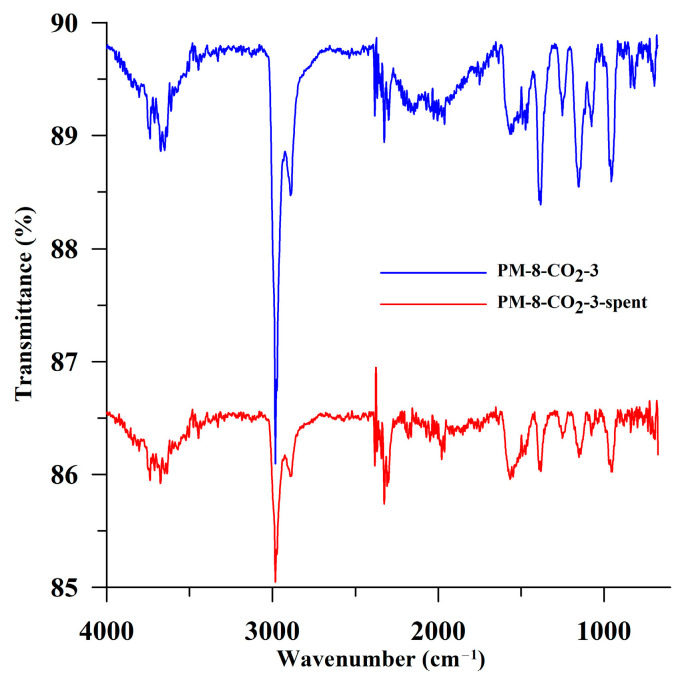
FTIR spectra of the PM-8-CO_2_-3 biocarbon before and after the MO adsorption process.

To determine changes in surface chemistry induced by the adsorption process, a comparison was made between the FTIR spectra of the biocarbon activated with CO_2_ for 3 h before contact with methyl orange and after its adsorption (Figure 10). The comparison of the FTIR spectra indicates that the adsorption process does not alter the overall shape of the spectrum, but primarily affects the relative intensities and shapes of the bands. This suggests that the fundamental chemical framework of the adsorbent is preserved. After adsorption, the most pronounced changes are observed in the regions around 3100–2800 cm^−1^ and 1600–1000 cm^−1^, where the development and increase in complexity of the signals can be seen. This can indicate overlap between adsorbate and adsorbent bands, as well as partial shielding of the biocarbon surface groups by the layer of adsorbed dye, rather than the simple disappearance of specific chemical moieties. This interpretation is consistent with reports for the methyl orange–based systems, in which adsorption was accompanied by preservation of the main spectral features of the adsorbent together with appearance or enhancement of bands associated with the presence of MO, particularly in the region of vibrations attributed to the aromatic systems, azo groups, and sulfonate moieties [43,44]. Consequently, the obtained results can be regarded as confirmation of effective binding of methyl orange on the surface of PM-8-CO_2_-3. The preservation of the overall spectral profile together with increased intensity of selected bands suggests that adsorption occurred mainly at the pre-existing active sites of the biocarbon, without substantial reconstruction of its chemical structure.

Table 10 presents a comparison of the MO adsorption capacities of the investigated biocarbons with those reported for other carbonaceous materials in the literature. The obtained results indicate that the prepared biocarbons exhibit favorable adsorption performance toward MO removal. In conclusion, pistachio shell waste can be considered a promising precursor for the production of activated biocarbons with significant potential for application as efficient adsorbents of MO in adsorption-based wastewater treatment processes.

## 4. Conclusions

The results of the studies indicate that the physical activation of biocarbon obtained from pistachio shells changes both its porous structure and surface chemistry, and the extent and nature of these changes substantially depend on the activating agent. CO_2_ activation promotes the production of materials with largely developed microporosity and a very large specific surface area, while steam activation results in the formation of a more open and hierarchical pore system, with a greater proportion of meso- and macropores. Of the tested samples, PM-8-CO_2_-3 and PM-8-H_2_O-2 were characterized by the most favorable textural parameters, with the vapor-activated material possessing a larger pore volume and a higher external surface area. Spectroscopic and chemical analyses confirmed that activation modifies not only the texture, but also the type of functional groups, as well as the elemental composition of biocarbons. In particular, steam activation resulted in a stronger reconstruction of the surface layer, an increase in the number of basic groups, and the acquisition of the most alkaline surface character. The Raman results also showed that activation does not result in simple graphitization, but leads to a complex reorganization of the carbon matrix, including partial ordering of local aromatic domains while maintaining numerous structural defects and the turbostratic nature of the materials. At the same time, an increase in the intensity of activation decreased the thermal stability of biocarbons, indicating that the development of porosity occurs at the expense of the degree of condensation of the carbon matrix.

The adsorption studies proved that the obtained biocarbons are effective adsorbents of methyl orange. The better efficiency of the PM-8-H_2_O-2 sample, despite a smaller proportion of micropores, indicates that the adsorption of methyl orange depends not only on the specific surface area but also on the presence of wider pores and a more accessible porous structure, facilitating mass transport. The adsorption kinetics is best described by the pseudo-second-order model, while studies of intra-particle diffusion proved that diffusion in pores was not the only step limiting the speed of the process, and that transport in the boundary layer also played an important role. The equilibrium data were best described by the Freundlich model, which indicates the heterogeneous nature of the surface and the possibility of multilayer adsorption. Thermodynamic analysis confirmed that the adsorption of methyl orange on the studied biocarbons is spontaneous and exothermic. The results prove that pistachio shells are a promising precursor for the production of highly efficient carbon adsorbents, and steam activation is particularly effective in the production of biocarbons intended for the removal of large anionic dyes from aqueous solutions.

## Figures and Tables

**Table 1:** Textural characteristics of the obtained activated biocarbons.

Sample	*S*_BET_	*S*_micro_	*S*_ext_	%*S*_micro_	*V*_p_	*V*_micro_	Δ*V*	*R*_av_	*V*_total_	*V*_macro_	*ρ*_b_
PM-8	0.5	n.d.	n.d.	n.d.	n.d.	n.d.	n.d.	n.d.	0.7353	n.d.	0.6133
PM-8-CO_2_-1	974	843	131	86	0.419	0.335	0.084	0.86	1.3491	0.9301	0.5219
PM-8-CO_2_-2	1270	1027	243	81	0.585	0.415	0.170	0.92	1.3928	0.8078	0.4186
PM-8-CO_2_-3	1478	1056	422	71	0.730	0.432	0.298	0.99	1.6542	0.9242	0.3898
PM-8-H_2_O-1	1177	854	323	73	0.684	0.352	0.333	1.16	1.8299	1.1459	0.3883
PM-8-H_2_O-2	1317	513	804	39	1.057	0.212	0.845	1.60	3.1259	2.0689	0.2569

Note: *S*_BET_—the specific surface area [m^2^ g^−1^]; *S*_micro_—specific surface area of micropores [m^2^ g^−1^]; *S*_ext_—outer surface area [m^2^ g^−1^]; %*S*_micro_—share of micropore area [%]; *V*_p_—total volume of sorption pores [cm^3^ g^−1^]; *V*_micro_—volume of micropores [cm^3^ g^−1^]; Δ*V*—the volume of meso- and macropores [cm^3^ g^−1^]; *R*_av_—the average pore radius [nm]; *V*_total_—the total volume of pores [cm^3^ g^−1^]; *V*_macro_—the volume of macropores [cm^3^ g^−1^]; *ρ*_b_—the bulk density [g cm^−3^]; n.d.—no data.

**Table 2:** The surface functional groups and pH of the tested biocarbons.

Sample	Basic Groups [mmol g^−1^]	Acidic Groups [mmol g^−1^]	Surface pH
PM-8	0.2394 ± 0.0648	0.1186 ± 0.0095	9.89 ± 0.0153
PM-8-CO_2_-1	0.8095 ± 0.0134	0.1813 ± 0.0561	10.26 ± 0.0473
PM-8-CO_2_-2	1.0629 ± 0.0251	0.1647 ± 0.0204	10.30 ± 0.0252
PM-8-CO_2_-3	1.2186 ± 0.0809	0.1392 ± 0.0339	10.35 ± 0.0551
PM-8-H_2_O-1	1.1123 ± 0.0508	0.0495 ± 0.0374	10.24 ± 0.0300
PM-8-H_2_O-2	2.0477 ± 0.0276	0.0019 ± 0.0001	10.59 ± 0.0755

**Table 3:** The results of elemental CHN analysis of selected biocarbons.

Sample	C% ± S.D.	H% ± S.D.	N% ± S.D.
PM-8	95.975 ± 0.166	0.103 ± 0.055	0.804 ± 0.026
PM-8-CO_2_-3	94.441 ± 1.168	0.127 ± 0.067	0.429 ± 0.076
PM-8-H_2_O-2	91.634 ± 1.393	0.406 ± 0.078	0.106 ± 0.015

Note: S.D.—standard deviation.

**Table 4:** The elemental composition (%*w*/*w*) of the tested biocarbons.

	PM-8	PM-8-CO_2_-1	PM-8-CO_2_-2	PM-8-CO_2_-3	PM-8-H_2_O-1	PM-8-H_2_O-2
C	92.96 ± 1.79	93.44 ± 1.91	93.11 ± 1.91	88.91 ± 2.02	87.52 ± 2.09	85.84 ± 2.00
O	4.45 ± 1.34	4.26 ± 1.35	4.04 ± 0.71	6.85 ± 0.49	7.21 ± 1.31	7.63 ± 1.22
Na	0.97 ± 0.20	0.55 ± 0.19	0.60 ± 0.17	0.22 ± 0.03	0.48 ± 0.20	0.20 ± 0.04
Cl	0.25 ± 0.21	0.18 ± 0.14	0.16 ± 0.11	0.07 ± 0.05	0.14 ± 0.15	0.05 ± 0.02
K	0.54 ± 0.40	0.58 ± 0.48	0.74 ± 0.44	0.61 ± 0.11	1.63 ± 1.30	1.27 ± 0.30
Sn	0.22 ± 0.05	0.25 ± 0.12	0.40 ± 0.43	0.92 ± 0.61	0.47 ± 0.32	1.79 ± 0.65
Ca	0.31 ± 0.25	0.46 ± 0.42	0.61 ± 0.58	1.77 ± 0.91	0.49 ± 0.19	1.97 ± 0.46
Fe	0.11 ± 0.09	0.08 ± 0.02	0.09 ± 0.08	0.11 ± 0.04	0.07 ± 0.02	0.10 ± 0.03
a *	2.40	2.09	2.59	3.69	3.28	5.39
b *	0.25	0.24	0.29	0.46	0.32	0.62

Note: a *—the sum of the proportion of elements (Na, Cl, K, Sn, Ca, and Fe) with the content > 0.1%; b *—the sum of the proportion of elements (Mg, Al, Si, P, S, and F) with the content < 0.1%.

**Table 5:** Raman spectroscopy data: peak positions (ν_D_, ν_G_), graphite peak (G), defect disorder peak (D), degree of graphitization using the ratio of the defect disorder peak intensity (I_D_/I_G_), crystallinity of biocarbon (K), factor (R^2^).

Sample	*ν*_D_ [cm^−1^]	*D*	*ν*_G_ [cm^−1^]	*G*	*I*_D_/*I*_G_	*K* [%]	*R* ^2^
PM-8	1318	1333	1588	981	1.49	42.4	0.9915
PM-8-CO_2_-1	1300	3890	1594	3059	1.27	42.4	0.9885
PM-8-CO_2_-2	1298	2824	1593	2097	1.35	42.6	0.9889
PM-8-CO_2_-3	1304	1679	1591	1156	1.45	40.8	0.9880
PM-8-H_2_O-1	1294	5447	1593	4679	1.16	46.2	0.9884
PM-8-H_2_O-2	1295	3579	1592	2965	1.21	45.3	0.9886

**Table 6:** The volatile matter, ash, and fixed-carbon contents.

Sample	%*VC*	%*FC*	%*A*
PM-8	5.75	89.82	4.43
PM-8-CO_2_-1	5.46	87.73	6.81
PM-8-CO_2_-2	8.91	83.03	8.06
PM-8-CO_2_-3	11.43	75.41	13.16
PM-8-H_2_O-1	9.34	85.14	5.52
PM-8-H_2_O-2	20.54	52.8	26.66

Note: %*VC*—the carbon content in the form of volatile compounds; %*FC*—the carbon content in solid form; %*A*—the ash content.

**Table 7:** The kinetic parameters for the MO adsorption on the tested biocarbons.

Model	Kinetic Model Equation	Parameter	Biocarbon	
			PM-8-CO_2_-3	PM-8-H_2_O-2
PFO	$\ln\left(q_{e}-q_{t}\right)=\ln q_{e}-k_{1}t$	*q*_e_ (mg g^−1^)	74.32	138.70
		*k*_1_ min^−1^	0.0063	0.0013
		*R* ^2^	0.9933	0.9380
PSO	$\frac{t}{q_{t}}=\frac{1}{k_{2}q_{e}^{2}}+\frac{t}{q_{e}}$	*q*_e_ (mg g^−1^)	204.08	384.62
		*k*_2_ (g mg^−1^ min^−1^)	0.00018	0.000045
		*R* ^2^	0.9996	0.9994
IPD	$q_{t}=k_{\mathrm{id}\;}t^{1/2}+c$	*k*_i_ (mg g^−1^ min^−0.5^)	3.22	6.77
		*C* (mg g^−1^)	121.55	188.76
		*R* ^2^	0.8695	0.9656
		*q*_e,exp_ (mg g^−1^)	195.63	394.66

Note: *k*_1_—the pseudo-first-order rate constants (min^−1^); *k*_2_—the pseudo-second-order rate constants (g mg^−1^ min^−1^); *t*—the time of contact between the adsorbent and adsorbate (min); *q*_e_—the adsorption value after equilibrium stabilization (mg g^−1^); *q*_t_—the adsorption value in given time *t* (mg g^−1^), respectively; *k*_id_—the intra-particle diffusion rate constant (mg g^−1^ min^−0.5^); and *c*—the intercept, which represents the thickness of the boundary layer (mg g^−1^).

**Table 8:** The isotherm parameters for the MO adsorption on the tested adsorbents.

Isotherm	Parameter	Biocarbon					
		PM-8-CO_2_-3			PM-8-H_2_O-2		
	Temperature (°C)	10	20	30	10	20	30
Langmuir	*K*_L_ (L mg^−1^)	0.0108	0.0064	0.0031	0.1407	0.0931	0.0539
	*q*_m_ (mg g^−1^)	353.10	367.27	413.92	519.40	518.83	521.18
	*R* ^2^	0.9680	0.9892	0.9897	0.8649	0.9043	0.9676
Freundlich	*K*_F_ (mg^1 − 1/n^ L^1/n^ g^−1^)	41.062	27.003	11.008	166.60	145.31	131.28
	*n*	0.317	0.371	0.497	0.209	0.232	0.239
	*R* ^2^	0.9970	0.9979	0.9988	0.9895	0.9880	0.9938
Langmuir–Freundlich	*K*_LF_ (L mg^−1^)	0.0058	0.0019	0.0024	0.1004	0.0579	0.0355
	*q*_m_ (mg g^−1^)	443.40	562.39	458.60	596.89	609.23	605.83
	*n*	0.656	0.620	0.886	0.564	0.591	0.622
	*R* ^2^	0.9926	0.9936	0.9924	0.8901	0.9357	0.9886
Temkin	*b*_T_ (J mol^−1^ g mg^−1^)	37.546	32.587	28.404	31.057	30.913	29.389
	*K*_T_ (L mg^−1^)	0.173	0.074	0.0350	2.8819	2.491	1.292
	*R* ^2^	0.9841	0.9934	0.9877	0.9875	0.9844	0.9875
	*q*_m,exp_	342.02	327.27	318.11	567.33	563.40	540.02

**Table 9:** The thermodynamic parameters for MO adsorption on the studied biocarbons.

Biocarbon	*T* (K)	∆*G* (kJ mol^−1^)	Δ*H* (kJ mol^−1^)	∆*S* (J K^−1^ mol^−1^)
PM-8-CO_2_-3	283	−19.22	−44.39	−88.59
	293	−18.64		
	303	−17.44		
PM-8-H_2_O-2	283	−25.27	−34.15	−31.16
	293	−25.15		
	303	−24.64		

**Table 10:** The comparison of the maximum MO adsorption capacities of the examined biochars with those reported for various carbon-based adsorbents.

Carbon Material	*S*_BET_ (m^2^ g^−1^)	Adsorption Capacity (mg g^−1^)	Ref.
PM-8-CO_2_-3	1478	342.02–318.11	This study
PM-8-H_2_O-2	1317	567.40–540.02	This study
DPB ^1^	-	461	[45]
PC ^2^	1714.5	109.5	[46]
ABPB ^3^	437.34	220	[47]
AC ^4^	-	129.3	[48]
CAC ^5^	714	107.53	[49]
LBC-800 ^6^	693	449	[50]
PPAB ^7^	75.32	163.2	[44]
PPC ^8^	877.3	239.1	[51]
MZ-biochar ^9^	1085	16.27	[52]
AC ^10^	835.2	244–333	[53]

Note: ^1^ biochar derived from date palm petioles; ^2^ KOH-activated pine cone biochar; ^3^ CO_2_-activated biocarbon derived from birchwood pellets; ^4^ commercial activated carbon; ^5^ H_3_PO_4_-activated corncob carbon; ^6^ multi-porous biochar derived from lotus biomass; ^7^ H_3_PO_4_-activated biochar derived from pomelo peel waste; ^8^ H_3_PO_4_-activated biochar derived from pomelo peel; ^9^ ZnCl_2_-activated mandarin peel biochar; ^10^ KOH-activated biochar derived from rice straw.

## Data Availability

The original contributions presented in this study are included in the article. Further inquiries can be directed to the corresponding author.
